# Supplementary material for: Improving AlphaFold Predicted Contacts for Alpha-Helical Transmembrane Proteins Using Structural Features
Source: Int J Mol Sci. 2024 May 11;25(10):5247. doi: 10.3390/ijms25105247 (PMC11121315; doi:10.3390/ijms25105247)
Supplement: Supplementary file 1 [file ijms-25-05247-s001.zip › ijms-2986103-supplementary.pdf]

Supplementary file 1 - Improving AlphaFold predicted contacts in alpha-helical transmembrane proteins structures using structural features

Aman Sawhney[1], Jiefu Li[2], Li Liao[1*]

[1*]Department of Computer and Information Sciences, University of Delaware, Smith Hall, 18 Amstel Avenue, 19716, DE, United States.
[2]School of Optical-Electrical and Computer Engineering, University of Shanghai for Science and Technology, 516 Jun Gong Road, Shanghai 200093, P. R. China.

*Corresponding author(s). E-mail(s): liliao@udel.edu;
Contributing authors: asawhney@udel.edu; lijiefu@usst.edu.cn;

1 Dataset - Experimentally determined structures

The following sequences were removed from the datasets:-

- S_L dataset - Sequences '5yi2B', '5lkiA', '5bw8D' in the S_L dataset have no positive inter-helical contacts, which would have led to Recall score being undefined, consequently we removed them from the dataset.
- S_{M1} dataset - For sequences '4p79A', '4qtnA', '4f35B' in the S_{M1} dataset, some of the residue positions annotated to be in TM zone don't match with the positions that Sun et. al [1] predicted on hence they were removed.
- S_{M2} dataset - For sequences '2rh1A', '3ukmA', '3m73A', '3m7lA in the S_{M2} dataset, some of the residue positions annotated to be in TM zone don't match with the positions that Sun et. al [1] predicted on hence they were removed.

With a final total of 162 sequences in the S_L dataset, 40 sequences in the S_{M2} dataset and 54 sequences in the S_{M1} dataset.

2 Dataset - Alphafold predicted structures

AlphaFold DB provides predicted structures for over 200 million protein sequences in the UniProt [2] reference proteome [3, 4]. These structures can be accessed via the protein chain's UniProtKB ID [2], and the 3-d coordinates for each residue's heavy atoms are available in PDB atomic coordinate format. We relied on Research Collaboratory for Structural Bioinformatics protein data bank (RCSB PDB [1]) [5, 6] to map the PDB ID of every chain in the DeepHelicon dataset to UniProtKB ID. If a match was found, the corresponding predicted structure was accessed via AlphaFold DB. For several protein chains, an integer offset to PDB positions in the DeepHelicon dataset is needed to sequentially align them with Alphafold structures, as is also reported in Faezov et. al [7]. In case a UniProtKB ID match was not found in RCSB PDB or the sequences from UniProt and DeepHelicon dataset matched partially i.e. all positions annotated to be in TM zones were not contiguously included, then the chain was removed from the dataset. This process leads to a final total of 154 sequences in the S_L dataset, 34 sequences in the S_{M2} dataset and 49 sequences in the S_{M1} dataset. In the subsequent subsections we explain in some detail the changes that were made to each dataset i.e. the cases when a sequence was removed or an integer offset was added.

2.1 S_L dataset

The changes for the S_L dataset are summarized in Table 1.

1. **1aigL** - Uniprot reports sequence match with PDB sequence for positions 2-282 (PDB sequence indices). Subtracting 1 from AlphaFold2 sequence positions will sequentially align the structures.
2. **2bhwA** - Uniprot reports sequence match with PDB sequence for positions 38-269 (PDB sequence indices), remaining positions are outside the TM zone. Subtracting 37from AlphaFold2 sequence positions will sequentially align the structures.
3. **2c3eA** - Uniprot reports sequence match with PDB sequence for positions 2-298 (PDB sequence indices). Subtracting 1 from AlphaFold2 sequence positions will sequentially align the structures.
4. **2priA** - Uniprot reports sequence match with PDB sequence for positions 2-843 (PDB sequence indices). Subtracting 1 from AlphaFold2 sequence positions will sequentially align the structures.
5. **3abvC** - Uniprot reports sequence match with PDB sequence for positions 30-169 (PDB sequence indices). Subtracting 26 from AlphaFold2 sequence positions will sequentially align the structures.
6. **3abvD** - Uniprot reports sequence match with PDB sequence for positions 57-159 (PDB sequence indices), remaining positions are outside the TM zone. Subtracting 23 from AlphaFold2 sequence positions will sequentially align the structures.
7. **3a3yA** - Uniprot reports a sequence match with PDB sequence for positions 1-1028 (entire sequence). PDB IDs in DeepHelicon dataset start at -4. Subtracting 5 from AlphaFold2 sequence positions will sequentially align the structures.

[1] RCSB.org

8. **3dh4A** - Uniprot reports sequence match with PDB sequence for positions 47-543 (PDB sequence indices), this matches DeepHelicon dataset. However, PDB IDs 8-19 in the DeepHelicon dataset are annotated as TM domain. Hence, removing this sequence.
9. **3m71A** - Uniprot reports sequence match with PDB sequence for positions 15-328 (PDB sequence indices), remaining positions are outside the TM zone. Subtracting 14 from AlphaFold2 sequence positions will sequentially align the structures.
10. **3zccA** - There are 2 matching entries in Uniprot O28769(unreviewed) and P0AEJ4(reviewed). We chose the reviewed entry. Uniprot reports sequence match with PDB sequence for positions 328-387 (PDB sequence indices), this matches PDB positions 229-288 in the DeepHelicon dataset. Adding 99 to AlphaFold2 sequence positions will sequentially align the structures.
11. **4a97A** - Uniprot reports sequence match with PDB sequence for positions 11-316 (PDB sequence indices). PDB in the DeepHelicon dataset start at 11, remaining positions are outside the TM zone. There is an additional residue at PDB id 154 in the DeepHelicon dataset. Adding 1 to AlphaFold2 sequence positions will sequentially align the structures.
12. **4bpdA** - Uniprot reports sequence match with PDB sequence for positions 2-122 (PDB sequence indices), remaining positions are outside the TM zone. PDB ids in DeepHelicon dataset start at -8. Subtracting 1 from AlphaFold2 sequence positions will sequentially align the structures.
13. **4g7vS** - Uniprot reports sequence match with PDB sequence for positions 11-182 (PDB sequence indices), remaining positions are outside the TM zone. PDB ids in DeepHelicon dataset start at 79. Adding 6 to AlphaFold2 sequence positions will sequentially align the structures.
14. **4jkvA** - Two Uniprot matches were found - P0ABE7 (length 128) and Q99835 (length 787), both are reviewed. We chose the Q99835 since it matches more residues. Uniprot reports sequence match with PDB sequence for positions 190-455 (PDB sequence indices), this would miss a few TM domains. Hence, removing the sequence.
15. **4jtaB** - Two Uniprot matches were found - P15387 (length 857) and P63142 (length 499). We chose the P63142 since it matches more residues. Uniprot does not report a sequence match with PDB sequence. From visual inspection, positions 1-266 and 303-499 match, this would miss two TM domains. Hence, removing the sequence.
16. **4phzA** - No Uniprot match was found for this sequence in RCSB hence, removing the sequence.
17. **4u1wA-** Uniprot does not report a sequence match with PDB sequence. From visual inspection, positions 25-847 match. Subtracting 21 from AlphaFold2 sequence positions will sequentially align the structures. Remaining residues and any irregularities are outside TM domains
18. **4wd7A** - No Uniprot match was found for this sequence in RCSB hence, removing the sequence.
19. **5a1sA** - No Uniprot match was found for this sequence in RCSB hence, removing the sequence.

20. **5a44A** - Uniprot reports sequence match with PDB sequence for positions 14-261 (PDB sequence indices), remaining positions are outside the TM zone. Subtracting 13 from AlphaFold2 sequence positions will sequentially align the structures.
21. **5iwkA** - Uniprot reports sequence match with PDB sequence for positions 41-709 (PDB sequence indices), remaining positions are outside the TM zone. Subtracting 40 from AlphaFold2 sequence positions will sequentially align the structures.
22. **5khnB** - No Uniprot match was found for this sequence in RCSB hence, removing the sequence.
23. **5lkiA** - This sequence is not present in AlphaFold DB, this is likely as the length of sequence is greater than 1280. More information can be found on https://alphafold.ebi.ac.uk/faq. Hence, removing the sequence.
24. **5l8rG** - Uniprot reports sequence match with PDB sequence for positions 62-158 (PDB sequence indices), remaining positions are outside the TM zone. Subtracting 4 from AlphaFold2 sequence positions will sequentially align the structures.
25. **5yi2B** - Uniprot reports sequence match with PDB sequence for positions 1-145 (PDB sequence indices), remaining positions are outside the TM zone. Subtracting 1 from AlphaFold2 sequence positions will sequentially align the structures.
26. **5zdhA** - Uniprot reports sequence match with PDB sequence for positions 41-686 (PDB sequence indices), remaining positions are outside the TM zone. Subtracting 40 from AlphaFold2 sequence positions will sequentially align the structures.
27. **6bhuA** - Uniprot reports sequence match with PDB sequence for positions 1-1530 (PDB sequence indices), this would miss the first two TM domains. Hence, removing the sequence.

2.2 S_{M1} dataset

The changes for the S_{M1} dataset are summarized in Table 2.

1. **1jb0L** - Uniprot reports sequence match with PDB sequence for positions 2-155 (PDB sequence indices), remaining positions are outside the TM zone. Subtracting 1 from AlphaFold2 sequence positions will sequentially align the structures.
2. **3wdoA** - No Uniprot match was found for this sequence in RCSB hence, removing the sequence.
3. **4bw5A** - Uniprot reports sequence match with PDB sequence for positions 62-335 (PDB sequence indices), remaining positions are outside the TM zone. Adding 5 from AlphaFold2 sequence positions will sequentially align the structures.
4. **4mesA** - No Uniprot match was found for this sequence in RCSB hence, removing the sequence.
5. **4phzB** - No Uniprot match was found for this sequence in RCSB hence, removing the sequence.
6. **4phzK** - No Uniprot match was found for this sequence in RCSB hence, removing the sequence.
7. **4q2eA** - Uniprot reports sequence match with PDB sequence for positions 1-270 (PDB sequence indices), remaining positions are outside the TM zone. Adding 20 from AlphaFold2 sequence positions will sequentially align the structures.

Table 1: AlphaFold predicted structures collection - S_L dataset

	PDB ID	Uniprot ID	Sequence length<br>DeepHelicon dataset	 RCSB	 Uniprot	Uniprot reported PDB match positions	Action
1	1aigL	P0C0Y8	281	281	282	2-282	Subtract 1 from AlphaFold2 sequence positions
2	2bhwA	P07371	232	232	269	38-269	Subtract 37 from AlphaFold2 sequence positions
3	2c3eA	P02722	297	297	298	2-298	Subtract 1 from AlphaFold2 sequence positions
4	2priA	P00489	842	842	843	2-843	Subtract 1 from AlphaFold2 sequence positions
5	3abvC	D0VWV4	140	140	169	30-169	Subtract 26 from AlphaFold2 sequence positions
6	3abvD	A5GZW8	103	103	159	57-159	Subtract 23 from AlphaFold2 sequence positions
7	3a3yA	Q4H132	1028	1028	1028	1-1028	Subtract 5 from AlphaFold2 sequence positions
8	3dh4A	P96169	530	530	543	47-543	Remove sequence
9	3m71A	P44741	314	314	328	15-328	Subtract 14 from AlphaFold2 sequence positions
10	3zccA	Multiple	114	114	-	-	Add 99 to AlphaFold2 sequence positions
11	4a97A	P0C7B7	307	307	321	11-316	Add 1 to AlphaFold2 sequence positions
12	4bpdA	P0ABN1	130	130	122	2-122	Subtract 1 from AlphaFold2 sequence positions
13	4g7vS	F6XHE4	185	185	570	83-254	Add 6 to AlphaFold2 sequence positions
14	4jkvA	Multiple	475	475	-	-	Remove this sequence
15	4jtaB	P15387	514	514	-	-	Remove sequence
16	4phzA	Uniprot match not found					Remove sequence
17	4u1wA	P19491	824	824	883	Not reported	Subtract 21 from AlphaFold2 sequence positions
18	4wd7A	Uniprot match not found					Remove sequence
19	5a1sA	Uniprot match not found					Remove sequence
20	5a44A	P02945	248	248	262	14-261	Subtract 13 from AlphaFold2 sequence positions
21	5iwkA	Q9R186	672	672	767	41-709	Subtract 40 from AlphaFold2 sequence positions
22	5khnB	Uniprot match not found					Remove sequence
23	5lkiA	Q9RN43	2516	2516	2516	1 -2516	Remove sequence (Already removed in previous section)
24	5l8rG	Q9S7N7	97	97	160	62-158	Subtract 4 from AlphaFold2 sequence positions
25	5yi2B	Q9CDU5	146	146	145	1-145	Subtract 1 from AlphaFold2 sequence positions<br>(Removed in previous section)
26	5zdhA	E3PJ86	646	646	686	41-686	Subtract 40 from AlphaFold2 sequence positions
27	6bhuA	Q8HXQ5	1659	1659	1530	1-1530	Remove sequence

8. **4qtnA** - Uniprot reports sequence match with PDB sequence for positions 28-263 (PDB sequence indices), remaining positions are outside the TM zone. Subtracting 25 from AlphaFold2 sequence positions will sequentially align the structures.
9. **5guwB** - Uniprot reports sequence match with PDB sequence for positions 1-466 (PDB sequence indices). There is an additional residue in Uniprot sequence at position 301, a simple offset would not sequentially align the structures, hence removing the sequence.
10. **6awfC** - Uniprot reports sequence match with PDB sequence for positions 1-359 (PDB sequence indices), remaining positions are outside the TM zone. Subtracting 1 from AlphaFold2 sequence positions will sequentially align the structures.
11. **6awfD** - Uniprot reports sequence match with PDB sequence for positions 1-359 (PDB sequence indices), remaining positions are outside the TM zone. Subtracting 1 from AlphaFold2 sequence positions will sequentially align the structures.

2.3 S_{M2} dataset

The changes for the S_{M2} dataset are summarized in Table 3.

1. **1xqfA** - Uniprot reports sequence match with PDB sequence for positions 23-428 (PDB sequence indices), remaining positions are outside the TM zone. Subtracting 22 from AlphaFold2 sequence positions will sequentially align the structures.
2. **2rh1A** - Uniprot does not report a sequence match with PDB sequence. From visual inspection, positions 1-230 match. This would exclude a few TM domains hence, removing the sequence.
3. **2wsc2** - Uniprot reports sequence match with PDB sequence for positions 1-269. The sequence includes in DeepHelicon dataset matches positions 94-269. Visual inspection of experimentally determined (PDBTM) structure and AlphaFold2 structure reveal 3 helices in both. While DeepHelicon dataset annotates 2 helices. It is likely that the annotations were updated, hence remove this sequence as a missing helix will lead to inaccurate reporting of a lower AlphaFold2 performance.
4. **2xq2A** - Uniprot does not report a sequence match with PDB sequence. From visual inspection, positions 1- 543 match. This would exclude the last TM domain hence, removing the sequence.
5. **2zxeA** - Uniprot reports a sequence match with PDB sequence for positions 1-1028 (entire sequence). PDB IDs in DeepHelicon dataset start at -4. Subtracting 5 from AlphaFold2 sequence positions will sequentially align the structures.
6. **3b9wA** - Uniprot reports a sequence match with PDB sequence for positions 1-450. There is a discrepancy of 7 residues within TM domain hence, removing the sequence.
7. **3eamA** - Uniprot reports a sequence match with PDB sequence for positions 44-359. PDB ID 44 according to Uniprot matches PDB ID 2 in DeepHelicon dataset. Subtracting 42 from AlphaFold2 sequence positions will sequentially align the structures.
8. **3rkoL** - No Uniprot match was found for this sequence in RCSB hence, removing the sequence.

Table 2: AlphaFold predicted structures collection - S_{M1} dataset

	PDB ID	Uniprot ID	Sequence length			Uniprot reported PDB match positions	Action
			DeepHelicon dataset	RCSB	Uniprot		
1	1jb0L	Q8DGB4	154	154	155	2-155	Subtract 1 from AlphaFold2 sequence positions
2	3wdoA	Uniprot match not found					Remove sequence
3	4bw5A	P57789	282	282	538	62-335	Add 5 toAlphaFold2 sequence positions
4	4mesA	Uniprot match not found					Remove sequence
5	4phzB	Uniprot match not found					Remove sequence
6	4phzK	Uniprot match not found					Remove sequence
7	4q2eA	Q9X1B7	290	290	270	1-270	Add 20 from AlphaFold2 sequence positions
8	4qtnA	D2ZZC1	244	244	263	28-263	Subtract 25 from AlphaFold2 sequence positions
9	5guwB	Q59647	465	465	466	1-466	Remove the sequence
10	6awfC	B7MKV9	130	130	131	1-131	Subtract 1 from AlphaFold2 sequence positions

9. **3rvyA** - Uniprot reports a sequence match with PDB sequence for positions 1-267. PDB 1 according to Uniprot matches PDB ID 1001 in DeepHelicon dataset. Adding 1000 to AlphaFold2 sequence positions will sequentially align the structures. Remaining residues are outside TM domains.
10. **4q2gB** - Uniprot reports a sequence match with PDB sequence for positions 1-270. Adding 20 to AlphaFold2 sequence positions will sequentially align the structures. Remaining residues are outside TM domains.
11. **4twdA** - Uniprot reports a sequence match with PDB sequence for positions 11-316. In DeepHelicon dataset, PDB ID starts at 11. However, there is an additional residue at position 154. Adding 1 to AlphaFold2 sequence positions will sequentially align the structures for all positions in the TM domains.
12. **4u1xC** Uniprot does not report a sequence match with PDB sequence. From visual inspection, positions 25-847 match. Subtracting 21 from AlphaFold2 sequence positions will sequentially align the structures. Remaining residues and any irregularities are outside TM domains.
13. **4wd8B** - No Uniprot match was found for this sequence in RCSB hence, removing the sequence.

3 Inter-helical tilt angle (θ)

For a residue pair, inter-helical tilt angle is defined as the angle between the helices the residues reside on [8]. In an α-helix, each main-chain $C = O$ and $N - H$ group is hydrogen bonded to a peptide bond four residues away i.e. $O(i)$ to $N(i + 4)$ (where i is the ith residue). The peptide planes are roughly parallel with the helical axis and the dipoles within the helix are aligned, i.e. all $C = O$ point in the same direction and all $N - H$ point in the other direction, while the side chains point outward from the helical axis (generally oriented towards the amino-terminal) [9]. This bond pattern is depicted in Fig. 1a.
Motivated by this observation, we compute any helical axis orientation by averaging the direction of $C(i) = O(i) - N(i+4)$ for all residues in the helix. The angle between the axes of two helices is the inter-helical tilt angle. Fig. 1b shows the inter-helical tilt angle between two helical axes. We use the Pymol package for these computations [10–12].

4 Relative residue angle (δ)

We defined a residue's plane as formed by the vector between C_α and N atom and the vector between C_α and C atom of the carboxyl group [13]. For a residue pair, we define the relative residue angle as the absolute angle between the surface-normals of the residue planes [14]. The angle is represented as δ in Fig. 2.

---

[2]First published in Lecture Notes in Computer Science [Volume 13920, Chapter 25] by Springer Nature Switzerland AG 2023
[3]First published in Lecture Notes in Computer Science [Volume 13920, Chapter 25] by Springer Nature Switzerland AG 2023

Table 3: AlphaFold predicted structures collection - S_{M2} dataset

	PDB ID	Uniprot ID	Sequence length			Uniprot reported PDB match positions	Action
			DeepHelicon dataset	RCSB	Uniprot		
1	1xqfA	P69681	418	418	428	23-428	Subtact 22 from AlphaFold sequence positions
2	2rh1A	P07550	500	500	413	Not reported	Remove sequence
3	2wsc2	Q41038	176	269	269	1-269	Remove sequence
4	2xq2A	P96169	593	593	543	Not reported	Remove sequence
5	2zxeA	Q4H132	1028	1028	1028	1-1028	Subtract 5 from AlphaFold sequence positions
6	3b9wA	Q82X47	407	407	425	25-425	Remove sequence
7	3eamA	Q7NDN8	317	317	359	44-359	Subtract 42 from AlphaFold sequence positions
8	3rkoL	Uniprot match not found					Remove sequence
9	3rvyA	A8EVM5	285	285	267	1-267	Add 1000 to AlphaFold sequence positions
10	4q2gB	Q9X1B7	290	290	270	1-270	Aadd 20 to AlphaFold sequence positions
11	4twdA	P0C7B7	307	307	321	11-316	Add 1 to AlphaFold sequence positions
12	4u1xC	P19491	824	824	883	Not reported	Subtract 21 from AlphaFold sequence positions
13	4wd8B	Uniprot match not found					Remove sequence

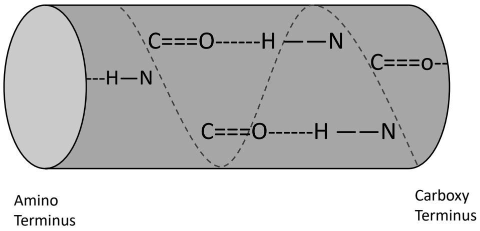

(a) Toilet roll representation of main chain hydrogen bonding in alpha-helix, adapted from [9]

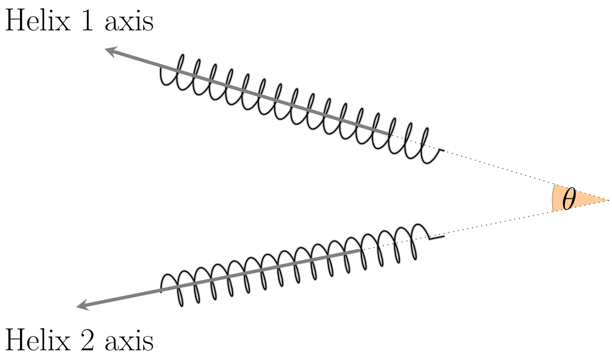

(b) Inter-helical tilt angle θ between the two helical axes

Fig. 1: Inter helical tilt angle [2]

5 Cross validation - random seeds

We use 5 fold cross validation in our experiments. During cross validation the dataset is split into 5 equal parts, in each fold the classifier is trained on 4 parts while tested on the remaining one. Since there are 5 folds, all samples are tested on once. In our implementation , which uses Scikit-learn [15], random seed is used to determine how the dataset is partitioned. Hence, it determines for a fold which sequences are used for training and which are tested on. We provide the seeds here for reproducibility. These were used for both coordinate as features (CF) and structurally derived features (SDF). Since, the cross validation experiment was repeated 5 times, 5 seeds for each dataset are reported in Table 4.

Table 4: Random seeds used in cross validation experiments.

Dataset	Iteration 1	Iteration 2	Iteration 3	Iteration 4	Iteration 5
S_L	3768687247	3744768744	3956695393	4112525849	2458923456
S_{M1}	2909617570	3986826679	4141477286	1589146018	1833799150
S_{M2}	4134222515	3265073376	2352221702	1732390130	2614245227

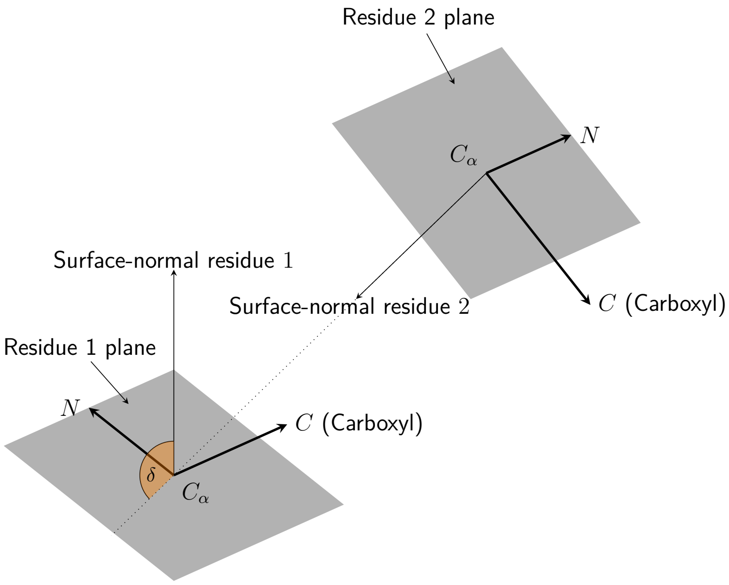

Fig. 2: Relative residue angle (δ) - Angle between the Surface-normals to the residue planes [3]

6 Classification results

6.1 Cross validation - L thresholds

We report the results for the cross validation experiments in terms of precision and recall in Table 5, where precision and recall are defined as follows :-

$$Precision = \frac{TP}{TP + FP} \quad \& \quad Recall = \frac{TP}{TP + FN} \tag{1}$$

where TP is the number of true positives, FP is the number of false positives and FN is the number of false negatives at a particular threshold. Precision and recall were computed for the top L, $L/2$, $L/5$, $L/10$ residue pair predictions where L denoted the total concatenated length of the TM helices for a sequence. For all metrics, we report the mean value across all sequences.

6.2 Held out results - L thresholds

We report the results for the cross validation experiments in terms of precision and recall in Table 6. For all metrics, we report the mean value across all sequences.

6.3 Held out results - per sequence results

Here we report the per sequence results for the held out experiments in terms of Average precision and AUC-ROC. We compare the performance of Structurally derived

features constructed using AlphaFold2 predicted structures (SDF +AF), coordinates as features from AlphaFold2 predicted structures (CF+ AF) and AlphaFold2 label annotations (AF2).

6.3.1 S_{M1} dataset

The results for S_{M1} dataset are reported in Table 7.

6.3.2 S_{M2} dataset

The results for S_{M2} dataset are reported in Table 8.

7 Classifier divergence

Here we design an experiment to assess why a classifier trained using SDF rather than CF can improve on AlphaFold's contact prediction performance. We train a classifier using features constructed from experimentally derived structures but during testing, only features constructed from AlphaFold predicted structures will be available to us. Consequently, classifier's testing performance depends on whether the feature distributions from the two sources are similar.
We assessed this via a second classifier's ability to differentiate between features generated using the two sources (AlphaFold & Experimental). Features constructed using experimentally determined structures are annotated with a label of 1, while those generated using AlphaFold's predicted structures are annotated as 0.
We also created a third set of features - Subtracted coordinates as features (SCF) i.e. the euclidean distance between the 3-d coordinates of corresponding heavy atoms. For a residue pair position (i, j), where i, j are amino acid sequence positions, s.t. $|i-j| > 5$ and i and j are on separate helices (inter-helical), we select a neighborhood window of size 3×3 around it. For each of the eight positions around (i, j) (excluding the center (i, j)), we constructed a feature vector of length 12 - consisting of difference between the x, y, z coordinates of the corresponding heavy atoms (N, C_α, O & C_β) from each residue in the pair of interest. We concatenated features for these eight neighboring positions to construct a feature vector of length 96 (12×8).
As is common practice, features from either feature set (SDF, CF or SCF) are first normalized to a $[0, 1]$ scale before being used for classification, such that $f^t_{i_{scaled}} = \frac{f^t_i - min(f_i)}{max(f_i) - min(f_i)}$ where f^t_i is the tth sample for the feature f_i, $max(.)$ and $min(.)$ compute the maximum and minimum observed value for the feature f_i and $f^t_{i_{scaled}}$ represents the scaled value of tth sample for the feature f_i.
We train a Logistic Regression classifier [16, 17] using SAGA solver [16, 18] and assess the performance on each dataset - S_L (154 sequences), S_{M1} (49 sequences) and S_{M2} (34 sequences) using 5 fold cross validation[19–21]. In each fold, 80% of randomly selected training sequences are used for training and 20% are held out for validation. We used the Scikit-learn package for our implementation [22].

Performance metrics

We measured Logistic Regression's ability to identify the sources of the structures using: -

1. Accuracy - Accuracy is the fraction of the correct predictions and is defined as

$$\texttt{Accuracy}(y, \hat{y}) = \frac{1}{N} \sum_{i=0}^{N-1} l \; ; \begin{cases} l = 1, & \text{if } \hat{y}_i = y_i \\ l = 0, & \text{otherwise} \end{cases} \tag{2}$$

   where $\hat{y}_i$ is the predicted label for the ith sample and y_i is the corresponding true label [23] and N is the total number of samples. If the features generated using Alphafold predicted structures and experimentally determined structures are entirely indistinguishable to the classifier, it will have an accuracy score 0.5.
2. Classifier divergence - Here our objective is to measure how distinguishable are the features generated using the two structural sources. For our purpose, an accuracy score A and $1 - A$ are equivalent. We define a metric Divergence that accounts for this.

$$Divergence \ = 2 \times |Accuracy - 0.5| \tag{3}$$

   If features generated using AlphaFold predicted structures and experimentally determined structures are indistinguishable, classifier's divergence score is 0.0. While if the classifier can perfectly distinguish between the two its divergence score is 1.0. Divergence score with variation in accuracy is depicted in Figure 3.

7.1 Results

In Table 9, we report a Logistic Regression classifier's ability to distinguish between features generated using AlphaFold and experimentally determined structures. We report average 5 fold cross validation performance in terms of accuracy and classifier divergence. CF constructed using AlphaFold and experimentally determined structures are very divergent or easy for the classifier to distinguish, with a divergence score of 0.49, 0.47 & 0.77 for S_L, S_{M1} & S_{M2} respectively. While SDF constructed using AlphaFold and experimentally determined structures are very hard for the classifier to distinguish with a divergence score 0.029, 0.0375 & 0.0314 for S_L, S_{M1} & S_{M2} datasets. SCF are far less divergent than CF with a divergence score of 0.06, 0.09 & 0.09 for S_L, S_{M1} & S_{M2} datasets.

8 Improvement example - 4g7vS

Phosphatidylinositol-3,4,5-trisphosphate 3-phosphatase (UniprotKB id - F6XHE4 in S_L dataset) from the organism - Transparent sea squirt (Ciona intestinalis), is a TM protein chain with 4 α-helices. It is involved in monoatomic ion channel activity and phosphorylation [24].

In this section, we illustrate how using a classifier trained on SDF from experimentally derived features can improve AlphaFold's predicted structure for 4g7vS. In Figure 4a, we depict a part of the interaction (183-194 & 161-165) between Helix 2 (PDB IDs

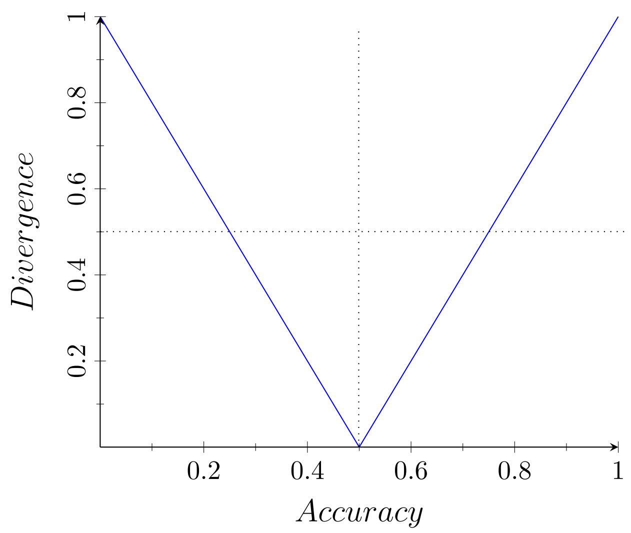

Fig. 3: Classifier divergence as a function of accuracy.

149-167) and Helix 3 (PDB IDs 183-204) as inferred from experimentally determined structure. In Figure 4b, the interaction inferred using AlphaFold's predicted structure are represented. AlphaFold's precision and recall [25] for this sequence are 0.6640 and 0.7442 respectively. AlphaFold incorrectly predicts 164 &186 and 164 & 190 as contact points. In Figure 4c, we represent the same part of the interaction between Helix 2 and Helix 3 when a classifier (5 fold cross validation experiment) trained using SDF is used to predict this sequence's contact map. We chose a threshold that maximized F1 score [26, 27], and using this threshold we make binary prediction for interactions, which achieves a precision and recall score of 0.7033 & 0.7442 respectively. Two false contact points between residue pairs 164-186 & 164-190 are correctly removed, at the cost of missing a true contact point between 165-183, resulting in an overall higher precision score.
The case study seems to suggest that using a residue pair's neighborhood structural information, the classifier is able to better account for atomic space constraints adjusting predicted contact propensities leading to a more accurate predicted structure.

9 Stratified by structure resolution

In this section we examine the effect of structure quality on the performance of the our method. First, we annotate every structure in our dataset as either 'high resolution' if the X-ray crystallography resolution is less than or equal to $2.5A^o$ or 'low resolution' if the resolution is greater than $2.5A^o$. Then we conduct three sets of experiments - 1) where we limit to just high resolution structures for both training and testing 2) where limit to just low resolution structures for training and testing 3) where we train and test on both high and low resolution structures with stratification (maintaining

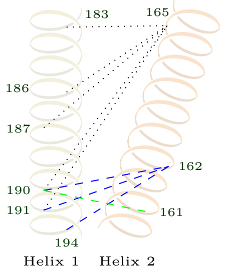

(a) Experimentally determined

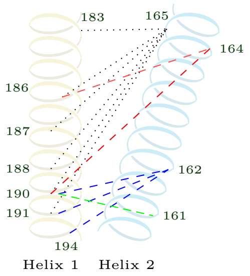

(b) Alphafold predicted

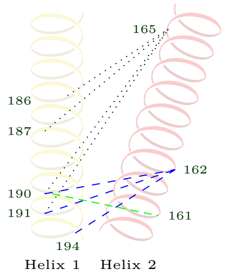

(c) Alphafold + SDF predicted

Fig. 4: Sequence 4g7vS (S_L dataset) partly represented. Contact points are indicated by connecting line segments.

the ratio of high resolution to low resolution structures in each fold) and report performance on structures of both qualities.
Overall, about a third of the structures in our dataset can be classified as high resolution using the aforementioned definition. Little less than a third of the structures in the S_L dataset and S_{M_1}; and about half the structures in S_{M_2} dataset are high resolution. We list the proportion of structures for each dataset in Table 10.

9.1 Cross validation - High resolution

In this experiment we limit to high resolution structures only i.e. with an X-ray crystallographic resolution of $2.5A^o$ or better. The proportion of high resolution structures for each dataset - S_L, S_{M_1} and S_{M_2} are reported in Table 10.
We evaluate our performance using 5 fold cross validation. During cross validation the dataset is split into 5 equal parts, in each fold the classifier is trained on 4 parts while tested on the remaining one. Since there are 5 folds, all samples are tested on once. In our implementation ,which uses Scikit-learn [15], random seed is used to determine how the dataset is partitioned. Hence, it determines for a fold which sequences are used for training and which are tested on. We provide the seeds here for reproducibility in Table 11.
When the dataset is limited to high resolution structures only, there is a performance improvement of 7.2%, 5.2% & 3.5% measured in terms of average precision

over AlphaFold2 for S_L, S_{M_1} & S_{M_2} datasets respectively. We report the classification performance in Table 12a.

9.2 Cross validation - Low resolution

In this experiment we limit to high resolution structures only i.e. with an X-ray crystallographic resolution of $2.5A^o$ or better. The proportion of high resolution structures for each dataset - S_L, S_{M_1} and S_{M_2} are reported in Table 10.
We evaluate our performance 5 fold cross validation in our experiments. During cross validation the dataset is split into 5 equal parts, in each fold the classifier is trained on 4 parts while tested on the remaining one. Since there are 5 folds, all samples are tested on once. In our implementation ,which uses Scikit-learn [15], random seed is used to determine how the dataset is partitioned. Hence, it determines for a fold which sequences are used for training and which are tested on. We provide the seeds here for reproducibility in Table 11.
When the dataset is limited to low resolution structures only, there is a performance improvement of 10.5%, 8.3% & 5.7% measured in terms of average precision over AlphaFold2 for S_L, S_{M_1} & S_{M_2} datasets respectively.We report the classification performance in Table 12b.

9.3 Cross validation - Stratified by resolution

In this experiment we use 5 fold stratified cross validation in our experiments. During cross validation the dataset is split into 5 equal parts, in each fold the classifier is trained on 4 parts while tested on the remaining one. Since there are 5 folds, all samples are tested on once. In stratified cross validation the percentage of samples from a class is maintained, here we use structure quality as the 'stratification variable'. Hence, in each fold the percentage of high resolution and low resolution structures is the same. In our implementation ,which uses Scikit-learn [15], random seed is used to determine how the dataset is partitioned. Hence, it determines for a fold which sequences are used for training and which are tested on. We provide the seeds here for reproducibility in Table 11.
When trained in a stratified manner, there is an improvement over AlphaFold 2 of 8.9%, 11% for high resolution & low resolution structures respectively for S_L dataset; 5.9% & 8.9% for high resolution & low resolution structures respectively for S_{M_1} dataset; 4.1, 7.4 for high resolution & low resolution structures respectively for S_{M_2}. We report the classification performance for S_L in Table 13a, S_{M_1}in Table 13b and S_{M_2}in Table 13c.

9.4 Discussion

We note that AlphaFold2's performance on low resolution structures is lower than on high resolution structures. Whether we train on high resolution or low resolution exclusively or together (stratified) an improvement over AlphaFold2 is observed. This performance improvement is higher for low resolution structures than for high resolution structures for all datasets. For S_L and S_{M_1} datasets the performance of

structurally derived features (SDF) on high and low resolution structures is very comparable (within $\pm 2\%$ in all 3 of the above mentioned experiments). The performance on low resolution structures is slightly higher which may be attributed to the higher proportion of low resolution structures. While in the case of S_{M_2} though there is a higher improvement in the performance on low resolution structures, the absolute performance on high resolution structures is much better (between 5-6% in all 3 experiments). This may be attributed to AlphaFold2's significantly better performance for high resolution structures in this case and to a nearly equal ratio of high and low resolution structures in this dataset.

Table (5) Classification performance - average over 5 fold Cross validation (repeated 5 times) in terms of precision and recall at L thresholds

Classifier	Structure source	Feature type	$L/10$		$L/5$		$L/2$		$L/1$	
			Precision	Recall	Precision	Recall	Precision	Recall	Precision	Recall
NN (upperbound)	Exp.	SDF	0.9859±0.0140	0.1313±0.0310	0.9725±0.0239	0.2211±0.0297	0.9463±0.0343	0.4657±0.0306	0.8538±0.0342	0.7474±0.0264
NN	AF	SDF	0.9585±0.0192	0.1245±0.0290	0.9454±0.0256	0.2119±0.0299	0.9114±0.0339	0.4446±0.0285	0.8174±0.0338	0.7157±0.0254
NN (upperbound)	Exp.	CF	0.9750±0.0201	0.1248±0.0274	0.9604±0.0291	0.2143±0.0277	0.9063±0.0371	0.4387±0.0265	0.7742±0.0362	0.6780±0.0243
NN	AF	CF	0.9511±0.0206	0.1211±0.0286	0.9372±0.0266	0.2085±0.0294	0.8834±0.0338	0.4255±0.0281	0.7574±0.0333	0.6629±0.0261

(a) S_L dataset

Classifier	Structure source	Feature type	$L/10$		$L/5$		$L/2$		$L/1$	
			Precision	Recall	Precision	Recall	Precision	Recall	Precision	Recall
NN (upperbound)	Exp.	SDF	0.9997±0.0014	0.0885±0.0145	0.9992±0.0017	0.1724±0.0275	0.9881±0.0101	0.4142±0.0608	0.9085±0.0491	0.7098±0.0515
NN	AF	SDF	0.9869±0.0132	0.0875±0.0144	0.9840±0.0158	0.1696±0.0269	0.9670±0.0179	0.4045±0.0582	0.8826±0.0474	0.6907±0.0520
NN (upperbound)	Exp.	CF	0.9861±0.0239	0.0877±0.0142	0.9779±0.0278	0.1682±0.0255	0.9340±0.0407	0.3850±0.0482	0.8068±0.0556	0.6306±0.0522
NN	AF	CF	0.9879±0.0147	0.0868±0.0141	0.9830±0.0163	0.1679±0.0262	0.9455±0.0261	0.3887±0.0534	0.8212±0.0453	0.6400±0.0539
DeepHelicon	-	-	0.8910±0.0413	0.0744±0.0108	0.8509±0.0443	0.1366±0.0176	0.7632±0.0479	0.2957±0.0307	0.6303±0.0469	0.4790±0.0414

(b) S_{M1} dataset

Classifier	Structure source	Feature type	$L/10$		$L/5$		$L/2$		$L/1$	
			Precision	Recall	Precision	Recall	Precision	Recall	Precision	Recall
NN (upperbound)	Exp.	SDF	0.9985±0.0038	0.0786±0.0155	0.9979±0.0030	0.1529±0.0282	0.9805±0.0227	0.3538±0.0420	0.9376±0.0389	0.6376±0.0468
NN	AF	SDF	0.9770±0.0226	0.0769±0.0151	0.9745±0.0224	0.1485±0.0260	0.9558±0.0345	0.3434±0.0417	0.9074±0.0389	0.6178±0.0520
NN (upperbound)	Exp.	CF	0.9697±0.0337	0.0750±0.0123	0.9592±0.0390	0.1438±0.0224	0.9240±0.0487	0.3277±0.0364	0.8368±0.0524	0.5656±0.0466
NN	AF	CF	0.9775±0.0293	0.0769±0.0152	0.9689±0.0250	0.1464±0.0238	0.9363±0.0357	0.3323±0.0349	0.8568±0.0406	0.5827±0.0431
DeepHelicon	-	-	0.9235±0.0336	0.0715±0.0128	0.8905±0.0318	0.1340±0.0230	0.7933±0.0412	0.2801±0.0350	0.6541±0.0441	0.4450±0.0470

(c) S_{M2} dataset

Exp - Experimentally derived structures
AF - AlphaFold predicted structures
SDF - Structurally derived featuresf

CF - Coordinates as features
NN - Neural network architecture presented in Main text

Table (6) Classification performance - held out datasets in terms of precision and recall at L thresholds

Classifier	Structure source	Feature type	$L/10$		$L/5$		$L/2$		$L/1$	
			Precision	Recall	Precision	Recall	Precision	Recall	Precision	Recall
NN (upperbound)	Exp.	SDF	1.0	0.0887	1.0	0.1730	0.9917	0.4171	0.9207	0.7204
NN	AF	SDF	0.9918	0.0880	0.9877	0.1702	0.9724	0.4069	0.8966	0.7024
NN (upperbound)	Exp.	CF	0.9878	0.0868	0.9769	0.1667	0.9334	0.3847	0.8005	0.6250
NN	AF	CF	0.9745	0.0860	0.9567	0.1628	0.9054	0.3719	0.7625	0.5982
DeepHelicon	-	-	0.8910	0.0745	0.8509	0.1368	0.7630	0.2960	0.6300	0.4791

(a) S_{M1} dataset

Classifier	Structure source	Feature type	$L/10$		$L/5$		$L/2$		$L/1$	
			Precision	Recall	Precision	Recall	Precision	Recall	Precision	Recall
NN (upperbound)	Exp.	SDF	1.0	0.0787	1.0	0.1534	0.9843	0.3562	0.9479	0.6455
NN	AF	SDF	0.9814	0.0776	0.9815	0.1491	0.9610	0.3453	0.9174	0.6246
NN (upperbound)	Exp.	CF	0.9964	0.0784	0.9762	0.1457	0.9396	0.3323	0.8351	0.5660
NN	AF	CF	0.9724	0.0768	0.9614	0.1458	0.9061	0.324	0.7943	0.5437
DeepHelicon	-	-	0.9235	0.0715	0.8905	0.1340	0.7932	0.2801	0.6541	0.4450

(b) S_{M2} dataset

Exp - Experimentally derived structures
AF - AlphaFold predicted structures
SDF - Structurally derived featuresf

CF - Coordinates as features
NN - Neural network architecture presented in Main text

Table 7: Per sequence results S_{M1} dataset

Sequence name	Average Precision			AUC-ROC		
	SDF+AF	CF+AF	AF2	SDF+AF	CF+AF	AF2
1xqfA	0.8994	0.7646	0.8523	0.9967	0.9898	0.9840
2cfqA	0.7844	0.6119	0.6326	0.9936	0.9852	0.9093
2jlnA	0.9266	0.7283	0.8318	0.9981	0.9890	0.9767
2nq2A	0.9607	0.7606	0.8854	0.9986	0.9902	0.9782
2r6gF	0.9291	0.7645	0.8215	0.9986	0.9930	0.9666
2r6gG	0.9363	0.8253	0.7867	0.9977	0.9937	0.9531
2w2eA	0.9514	0.8033	0.9019	0.9970	0.9893	0.9740
2wswA	0.9487	0.7445	0.9124	0.9988	0.9921	0.9802
2yevA	0.9520	0.7753	0.8661	0.9992	0.9954	0.9723
2yvxA	0.8670	0.8029	0.7042	0.9945	0.9882	0.9208
2z73A	0.9590	0.7615	0.9289	0.9982	0.9887	0.9823
2zxeA	0.9275	0.7308	0.8320	0.9979	0.9896	0.9443
2zy9A	0.8934	0.7571	0.7488	0.9939	0.9856	0.9370
3c02A	0.9679	0.8305	0.9491	0.9989	0.9920	0.9867
3ddlA	0.9358	0.7723	0.8479	0.9973	0.9880	0.9543
3eamA	0.9358	0.8029	0.9171	0.9972	0.9880	0.9879
3gd8A	0.9626	0.8537	0.9319	0.9985	0.9926	0.9865
3giaA	0.8930	0.7276	0.7451	0.9937	0.9897	0.9292
3hd6A	0.9600	0.7989	0.9040	0.9989	0.9927	0.9813
3k3fA	0.9567	0.8179	0.9601	0.9984	0.9916	0.9870
3klyA	0.9123	0.7660	0.8555	0.9955	0.9889	0.9656
3qe7A	0.6110	0.5132	0.5278	0.9758	0.9806	0.8905
3rvyA	0.8510	0.7853	0.8690	0.9949	0.9920	0.9592
3t9nA	0.9828	0.8554	0.9688	0.9994	0.9949	0.9996
3tijA	0.9601	0.7691	0.9176	0.9989	0.9917	0.9923
3usiA	0.9402	0.7577	0.8672	0.9982	0.9923	0.9646
3v5uA	0.9666	0.8064	0.9105	0.9991	0.9929	0.9791
4czbB	0.9353	0.7515	0.8229	0.9984	0.9923	0.9677
4hygA	0.9440	0.7814	0.7946	0.9981	0.9902	0.9444
4ikwA	0.9580	0.7674	0.9033	0.9993	0.9944	0.9807
4m5bA	0.9653	0.8197	0.9591	0.9985	0.9903	0.9874
4q2gB	0.9285	0.7964	0.8739	0.9981	0.9932	0.9805
4r0cB	0.9438	0.7665	0.8763	0.9986	0.9934	0.9649
4twdA	0.9543	0.6924	0.8341	0.9944	0.9762	0.9692
4u1xC	0.7902	0.8396	0.7134	0.9949	0.9965	0.9122

Table 8: Per sequence results S_{M2} dataset

Sequence name	Average Precision			AUC-ROC		
	SDF+AF	CF+AF	AF2	SDF+AF	CF+AF	AF2
1jb0L	0.9496	0.8778	0.9341	0.9947	0.9878	0.9790
2a06C	0.9742	0.7724	0.9150	0.9994	0.9929	0.9820
2a65A	0.9404	0.7683	0.8692	0.9986	0.9931	0.9656
2abmA	0.9640	0.8029	0.8992	0.9984	0.9883	0.9812
2aczC	0.9315	0.7711	0.8600	0.9968	0.9787	0.9821
2aczD	0.9178	0.8821	0.8553	0.9976	0.9944	0.9893
2axtB	0.9770	0.8290	0.8690	0.9994	0.9936	0.9743
2axtZ	1.0000	0.9382	0.9524	1.0000	0.9972	0.9986
2bs2C	0.9762	0.7382	0.9525	0.9984	0.9891	0.9861
2zuqA	0.9160	0.7892	0.7430	0.9858	0.9727	0.9032
3abkA	0.9575	0.7648	0.8861	0.9989	0.9932	0.9785
3b4rA	0.9387	0.7899	0.7987	0.9976	0.9917	0.9561
3mp7A	0.8597	0.6959	0.7066	0.9969	0.9900	0.9468
3o7pA	0.9470	0.7905	0.8664	0.9986	0.9930	0.9647
3tuiA	0.9386	0.8043	0.8249	0.9984	0.9930	0.9713
3ux4A	0.9396	0.7666	0.8159	0.9967	0.9881	0.9453
4a4mA	0.9538	0.7603	0.8598	0.9986	0.9883	0.9793
4bw5A	0.8962	0.5870	0.8489	0.9987	0.9903	0.9723
4dntA	0.9393	0.7154	0.7968	0.9983	0.9902	0.9425
4dxwA	0.8580	0.6686	0.7199	0.9920	0.9820	0.8768
4fc4A	0.9493	0.7659	0.9567	0.9985	0.9915	0.9900
4he8D	0.9592	0.8991	0.7404	0.9992	0.9979	0.9291
4he8F	0.9372	0.7787	0.8388	0.9986	0.9939	0.9589
4j05A	0.9100	0.7078	0.8057	0.9970	0.9888	0.9550
4kppA	0.9032	0.7516	0.7845	0.9963	0.9930	0.9478
4oqyA	0.9143	0.8200	0.7997	0.9969	0.9932	0.9251
4pgrA	0.7050	0.6341	0.6003	0.9560	0.9721	0.8609
4q2eA	0.9163	0.7841	0.8327	0.9975	0.9920	0.9659
4rp8A	0.9442	0.8009	0.9109	0.9983	0.9931	0.9768
4ryiA	0.9632	0.7785	0.8731	0.9984	0.9828	0.9572
4tquM	0.9564	0.7891	0.9413	0.9986	0.9902	0.9763
4xksA	0.9306	0.7197	0.8626	0.9955	0.9788	0.9641
4ymsD	0.9612	0.7968	0.8870	0.9986	0.9904	0.9726
5a8eA	0.9240	0.7490	0.8505	0.9981	0.9919	0.9711
5b57A	0.9476	0.7255	0.8718	0.9985	0.9912	0.9703
5c6nA	0.8470	0.7214	0.6172	0.9963	0.9931	0.9064
5doqA	0.9670	0.7635	0.9390	0.9988	0.9878	0.9820
5gufA	0.9609	0.8064	0.9030	0.9985	0.9866	0.9884
5jkiA	0.9323	0.8306	0.7074	0.9958	0.9871	0.8583
5kbwA	0.9365	0.8196	0.8741	0.9968	0.9854	0.9794
5l26A	0.9537	0.7456	0.8990	0.9990	0.9923	0.9869
5o0tA	0.9089	0.6827	0.7740	0.9960	0.9857	0.9357
5x5yG	0.7728	0.6941	0.5963	0.9926	0.9877	0.8948
5xjjA	0.9319	0.7746	0.8309	0.9984	0.9932	0.9605
5xu1M	0.8917	0.7575	0.6992	0.9918	0.9858	0.9187
6awfC	0.9571	0.7714	0.8866	0.9980	0.9884	0.9568
6awfD	0.8955	0.8371	0.8060	0.9963	0.9918	0.9819
6barA	0.8789	0.7059	0.8053	0.9657	0.9770	0.9313
6cb2A	0.9754	0.8544	0.8826	0.9991	0.9947	0.9736

Table 9: Classifier divergence - How well can a classifier differentiate between AlphaFold predicted and Experimental structures?

	S_L		S_{M1}		S_{M2}	
Features	Accuracy	Divergence	Accuracy	Divergence	Accuracy	Divergence
SDF[5]	0.5145 ± 0.0067	0.0290 ± 0.0133	0.5187 ± 0.0066	0.0375 ± 0.0132	0.5157 ± 0.0062	0.0314 ± 0.0125
CF[6]	0.7467 ± 0.0299	0.4934 ± 0.0597	0.7365 ± 0.0884	0.4731 ± 0.1768	0.8859 ± 0.0325	0.7717 ± 0.0651
SCF[7]	0.5315 ± 0.0231	0.0629 ± 0.0461	0.5456 ± 0.0320	0.0912 ± 0.0640	0.4559 ± 0.0381	0.0943 ± 0.0665

Table 10: Fraction of structures in each dataset by resolution.

Dataset	High resolution (%)	Low resolution (%)
Overall	33.04	66.96
S_L	28.94	71.04
S_{M1}	32.61	67.39
S_{M2}	51.43	48.57

Table 11: Random seeds used in cross validation experiments.

Dataset	Iteration 1
S_L	8844592
S_{M1}	245
S_{M2}	4249567

Table (12) Classification performance - average over 5 fold cross validation in terms of average precision. Dataset limited to structures of high or low resolution.

Classifier	Structure source	Feature type	Average Precision		
			S_L	S_{M_1}	S_{M_2}
NN (upperbound)	Exp.	SDF	0.9425 ± 0.01454	0.9373 ± 0.01098	0.9438 ± 0.00759
NN	AF	SDF	0.8722 ± 0.05019	0.8990± 0.03833	0.9255 ± 0.01989
AlphaFold2	-	-	0.8001	0.8471	0.8902

(a) Datasets filtered for high resolution structures only.

Classifier	Structure source	Feature type	Average Precision		
			S_L	S_{M_1}	S_{M_2}
NN (upperbound)	Exp.	SDF	0.9563 ± 0.00565	0.9430 ± 0.00486	0.9265 ± 0.01695
NN	AF	SDF	0.8946 ± 0.02181	0.9049 ± 0.01695	0.8635 ± 0.07272
AlphaFold2	-	-	0.7887	0.8216	0.8066

(b) Datasets filtered for low resolution structures only.

Table (13) Classification performance - average over 5 fold stratified cross validation in terms of average precision. Each stratified fold preserves the ratio of high resolution to low resolution structures. Results reported for each resolution separately.

Classifier	Structure source	Feature type	Average Precision	
			High resolution	Low resolution
NN (upperbound)	Exp.	SDF	0.9589 ± 0.00888	0.9587 ± 0.00482
NN	AF	SDF	0.8896 ± 0.06191	0.8987 ± 0.02435
AlphaFold2	-	-	0.8001	0.7887

(a) Stratified cross validation results for S_L dataset.

Classifier	Structure source	Feature type	Average Precision	
			High resolution	Low resolution
NN (upperbound)	Exp.	SDF	0.9495 ± 0.01760	0.9502 ± 0.00395
NN	AF	SDF	0.9064 ± 0.03580	0.9106 ± 0.02541
AlphaFold2	-	-	0.8471	0.8216

(b) Stratified cross validation results for S_{M_1} dataset.

Classifier	Structure source	Feature type	Average Precision	
			High resolution	Low resolution
NN (upperbound)	Exp.	SDF	0.9497 ± 0.00659	0.9444 ± 0.01172
NN	AF	SDF	0.9318 ± 0.01841	0.8804 ± 0.05391
AlphaFold2	-	-	0.8902	0.8066

(c) Stratified cross validation results for S_{M_2} dataset.

References

[1] Sun J, Frishman D. DeepHelicon: Accurate prediction of inter-helical residue contacts in transmembrane proteins by residual neural networks. Journal of Structural Biology. 2020;212(1):107574.

[2] UniProt Consortium T. UniProt: the Universal Protein knowledgebase in 2023. Nucleic Acids Research. 2023;51(D1):D523–D531.

[3] Alphafold DB.: AlphaFold Protein Structure Database. (Accessed on 05/23/2023). https://alphafold.ebi.ac.uk/.

[4] Varadi M, Anyango S, Deshpande M, Nair S, Natassia C, Yordanova G, et al. AlphaFold Protein Structure Database: massively expanding the structural coverage of protein-sequence space with high-accuracy models. Nucleic acids research. 2022;50(D1):D439–D444.

[5] Burley SK, Bhikadiya C, Bi C, Bittrich S, Chao H, Chen L, et al. RCSB Protein Data Bank (RCSB. org): delivery of experimentally-determined PDB structures alongside one million computed structure models of proteins from artificial intelligence/machine learning. Nucleic Acids Research. 2023;51(D1):D488–D508.

[6] Berman HM, Battistuz T, Bhat TN, Bluhm WF, Bourne PE, Burkhardt K, et al. The protein data bank. Acta Crystallographica Section D: Biological Crystallography. 2002;58(6):899–907.

[7] Faezov B, Dunbrack Jr RL. PDBrenum: A webserver and program providing Protein Data Bank files renumbered according to their UniProt sequences. PLoS One. 2021;16(7):e0253411.

[8] Lee HS, Choi J, Yoon S. QHELIX: a computational tool for the improved measurement of inter-helical angles in proteins. The protein journal. 2007;26(8):556–561.

[9] Cooper J.: Alpha-Helix Geometry Part. 2 — cryst.bbk.ac.uk. [Accessed 25-Jan-2022]. http://www.cryst.bbk.ac.uk/PPS95/course/3_geometry/helix2.html.

[10] Schrödinger, LLC. The AxPyMOL Molecular Graphics Plugin for Microsoft PowerPoint, Version 1.8; 2015.

[11] Schrödinger, LLC. The JyMOL Molecular Graphics Development Component, Version 1.8; 2015.

[12] Schrödinger, LLC. The PyMOL Molecular Graphics System, Version 1.8; 2015.

[13] Mahbub S, Bayzid MS. EGRET: Edge Aggregated Graph Attention Networks and Transfer Learning Improve Protein-Protein Interaction Site Prediction.

bioRxiv. 2021;p. 2020–11.

[14] Sawhney A, Li J, Liao L. Inter-helical residue contact prediction in α-helical Transmembrane proteins using structural features; 2023. In press, Lecture Notes in Bioinformatics (LNBI), 10th International Work-Conference on Bioinformatics and Biomedical Engineering (IWBBIO).

[15] Sklearn KFold.: sklearn.model_selection.KFold — scikit-learn 0.23.2 documentation. (Accessed on 07/30/2023). https://scikit-learn.org/0.23/modules/generated/sklearn.model_selection.KFold.html?highlight=kfold#sklearn.model_selection.KFold.

[16] Scikit-learn Logistic.: sklearn.linear_model.LogisticRegression — scikit-learn 1.2.2 documentation. (Accessed on 06/07/2023). https://scikit-learn.org/stable/modules/generated/sklearn.linear_model.LogisticRegression.html.

[17] Wikipedia Logistic.: Logistic regression - Wikipedia. (Accessed on 06/07/2023). https://en.wikipedia.org/wiki/Logistic_regression#References.

[18] Defazio A, Bach F, Lacoste-Julien S. SAGA: A fast incremental gradient method with support for non-strongly convex composite objectives. Advances in neural information processing systems. 2014;27.

[19] James G, Witten D, Hastie T, Tibshirani R. An introduction to statistical learning. vol. 112. Springer; 2013.

[20] Friedman J, Hastie T, Tibshirani R, et al. The elements of statistical learning. vol. 1. Springer series in statistics New York; 2001.

[21] Kohavi R, et al. A study of cross-validation and bootstrap for accuracy estimation and model selection. In: Ijcai. vol. 14. Montreal, Canada; 1995. p. 1137–1145.

[22] Pedregosa F, Varoquaux G, Gramfort A, Michel V, Thirion B, Grisel O, et al. Scikit-learn: Machine Learning in Python. Journal of Machine Learning Research. 2011;12:2825–2830.

[23] Scikit-accuracy.: 3.3. Metrics and scoring: quantifying the quality of predictions — scikit-learn 1.2.2 documentation. (Accessed on 06/07/2023). https://scikit-learn.org/stable/modules/model_evaluation.html#accuracy-score.

[24] Uniprot - 4g7vS.: Phosphatidylinositol-3,4,5-trisphosphate 3-phosphatase - Ciona intestinalis (Transparent sea squirt) — UniProtKB — UniProt. (Accessed on 06/10/2023). https://www.uniprot.org/uniprotkb/F6XHE4/entry#names_and_taxonomy.

[25] Wikipedia Precision.: Precision and recall - Wikipedia. (Accessed on 06/10/2023). https://en.wikipedia.org/wiki/Precision_and_recall.

[26] Wikipedia F-score.: F-score - Wikipedia. (Accessed on 06/10/2023). https://en.wikipedia.org/wiki/F-score.

[27] Sklearn F1.: sklearn.metrics.f1_score — scikit-learn 0.24.2 documentation. (Accessed on 06/10/2023). https://scikit-learn.org/0.24/modules/generated/sklearn.metrics.f1_score.html?highlight=f1%20score#sklearn.metrics.f1_score.
